# L‐Arginine‐Loaded Gold Nanocages Ameliorate Myocardial Ischemia/Reperfusion Injury by Promoting Nitric Oxide Production and Maintaining Mitochondrial Function

**DOI:** 10.1002/advs.202302123

**Published:** 2023-07-14

**Authors:** Zekun Wang, Nana Yang, Yajun Hou, Yuqing Li, Chenyang Yin, Endong Yang, Huanhuan Cao, Gaofei Hu, Jing Xue, Jialei Yang, Ziyu Liao, Weiyun Wang, Dongdong Sun, Cundong Fan, Lemin Zheng

**Affiliations:** ^1^ School of Life Sciences Anhui Agricultural University Hefei Anhui 230036 China; ^2^ School of Bioscience and Technology Weifang Key Laboratory of Animal Model Research on Cardiovascular and Cerebrovascular Diseases Weifang Medical University Weifang 261053 China; ^3^ Department of Neurology Second Affiliated Hospital Shandong First Medical University & Shandong Academy of Medical Sciences Taian Shandong 271000 China; ^4^ The Institute of Cardiovascular Sciences and Institute of Systems Biomedicine School of Basic Medical Sciences State Key Laboratory of Vascular Homeostasis and Remodeling NHC Key Laboratory of Cardiovascular Molecular Biology and Regulatory Peptides Beijing Key Laboratory of Cardiovascular Receptors Research Health Science Center Peking University Beijing 100191 China; ^5^ Department of Neurology China National Clinical Research Center for Neurological Diseases Beijing Tiantan Hospital Capital Medical University Beijing 100070 China

**Keywords:** L‐arginine, mitochondrial function, myocardial function, myocardial ischaemia/reperfusion injury, nitric oxide, reactive oxygen species

## Abstract

Cardiovascular disease is the leading cause of death worldwide. Reperfusion therapy is vital to patient survival after a heart attack but can cause myocardial ischemia/reperfusion injury (MI/RI). Nitric oxide (NO) can ameliorate MI/RI and is a key molecule for drug development. However, reactive oxygen species (ROS) can easily oxidize NO to peroxynitrite, which causes secondary cardiomyocyte damage. Herein, L‐arginine‐loaded selenium‐coated gold nanocages (AAS) are designed, synthesized, and modified with PCM (WLSEAGPVVTVRALRGTGSW) to obtain AASP, which targets cardiomyocytes, exhibits increased cellular uptake, and improves photoacoustic imaging in vitro and in vivo. AASP significantly inhibits oxygen glucose deprivation/reoxygenation (OGD/R)‐induced H9C2 cell cytotoxicity and apoptosis. Mechanistic investigation revealed that AASP improves mitochondrial membrane potential (MMP), restores ATP synthase activity, blocks ROS generation, and prevents NO oxidation, and NO blocks ROS release by regulating the closing of the mitochondrial permeability transition pore (mPTP). AASP administration in vivo improves myocardial function, inhibits myocardial apoptosis and fibrosis, and ultimately attenuates MI/RI in rats by maintaining mitochondrial function and regulating NO signaling. AASP shows good safety and biocompatibility in vivo. This findings confirm the rational design of AASP, which can provide effective treatment for MI/RI.

## Graphical abstract

A nanoplatform (AASP) for myocardial targeted delivery of nitric oxide (NO) is developed for the treatment of myocardial ischemia‐reperfusion injury (MI/RI). AASP blocks reactive oxygen species release by regulating the closure of the mitochondrial permeability transition pore via NO, preventing NO oxidation and improving mitochondrial function, thereby inhibiting myocardial apoptosis and fibrosis, ultimately reducing MI/RI in rats.

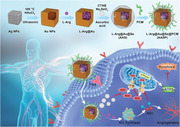

## 1 Introduction

Cardiovascular disease is the leading cause of death worldwide, and ischemic heart disease causes ≈50% of all deaths.^[^ ^1^ ^]^ Acute myocardial infarction due to narrowing or complete occlusion of the coronary arteries is the most common manifestation of ischemic heart disease. After a myocardial infarction, the damaged myocardium eventually undergoes a remodeling process that involves myocardial cell death, tissue fibrotic scarring and cardiac dilation, ultimately leading to heart failure.^[^ ^2^ ^]^ Although reperfusion of ischemic myocardial tissue is critical for patient survival, it also causes myocardial ischemia/reperfusion injury (MI/RI), which includes oxidative damage, cell death and inflammatory responses. Effective clinical treatments for these effects remain lacking.^[^ ^3^ ^]^

Recently, important progress has been made in the development of innovative therapeutic strategies for ischemic cardiomyopathy, including stem cell‐based regenerative therapy,^[^ ^4^ ^]^ cardiac patch therapy,^[^ ^5^ ^]^ and biomaterial‐based approaches.^[^ ^6^ ^]^ Nanotechnology holds great promise for the development of therapeutic strategies for ischemic cardiomyopathy. There has also been increasing interest in the development of targeted nanomedicines for postischemic cardiac injury.^[^ ^7^ ^]^ Gold nanocages are metal nanoparticles with hollow structures, mesoporous surfaces and plasmonic properties. Gold nanocages can be effectively used for the encapsulation, delivery and release of drug molecules under specific conditions.^[^ ^8^ ^]^ Such nanoparticles exhibit good light scattering and absorption capabilities in the near‐infrared region and are widely used in photoacoustic imaging and photothermal therapy research.^[^ ^9^ ^]^ PCM is a cardiomyocyte‐targeting peptide that was identified by phage display technology; PCM can specifically recognize tenascin X (TNX) on the surface of cardiomyocytes and has been widely used for modifying drugs and drug carriers.^[^ ^10^ ^]^ PCM‐modified gold nanocages are expected to be useful for drug molecule delivery and photoacoustic imaging targeting the myocardial region.

Nitric oxide (NO), which is a multifunctional signaling molecule, plays an important role in the regulation of cardiovascular homeostasis. L‐arginine (L‐Arg) is catalyzed by nitric oxide synthase in the presence of O_2_ and NADPH. NO can reduce MI/RI by relaxing vascular tone, inhibiting platelet aggregation, and regulating inflammatory responses.^[^ ^11^ ^]^ NO inhibits the activity of complex I in the mitochondrial electron transport chain, thereby limiting the generation of mitochondrial reactive oxygen species (ROS).^[^ ^12^ ^]^ This further prevents the opening of the mitochondrial permeability transition pore (mPTP) and reduces the release of cytochrome c and apoptosis of cardiomyocytes.^[^ ^13^ ^]^ However, NO rapidly reacts with excessive levels of ROS in myocardial tissue subjected to MI/RI, and this reaction generates peroxynitrite, which further damages the tissues.^[^ ^14^ ^]^ Therefore, enhancing ROS inhibition and NO generation is key for the success of this therapeutic strategy. Selenium has novel antioxidant properties and acts as a component of glutathione peroxidase. Selenium deficiency can lead to a significant decrease in glutathione peroxidase activity and result in increased oxidative stress.^[^ ^15^ ^]^ Nanoselenium (Se NPs) has been widely studied due to its high antioxidant activity and low toxicity and because it can be easily modified. Li et al. confirmed that 11‐mercapto‐1‐undecanol‐modified selenium nanoparticles had free radical scavenging activity and antagonized cisplatin‐induced nephrotoxicity.^[^ ^16^ ^]^ However, little information is available about the protective effect of Se NPs against MI/RI. Hence, the design of novel selenium nanocomposites for use in MI/RI therapy is of great importance.

**Figure 1:**
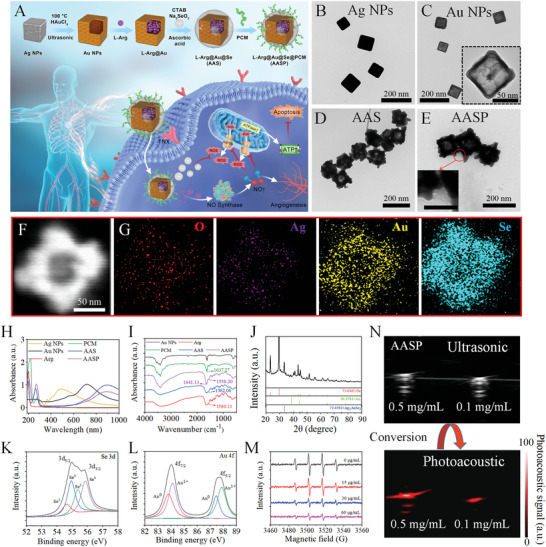
Synthesis and characterization. A) Synthesis route and mechanism underlying the effects. L‐Arg‐loaded gold nanocages ameliorate myocardial ischemia/reperfusion injury by promoting nitric oxide production and maintaining mitochondrial function. TEM images of Ag nanoparticles (B), Au nanocages (C), AAS (D) and AASP (E). F) SEM image of AASP. G) Element map image of AASP. H) UV‒vis‐NIR spectra of Ag NPs, Au NPs, L‐Arg, PCM, AAS and AASP. I) FTIR spectra of Au NPs, L‐Arg, PCM, AAS and AASP. J) XRD pattern of AASP powder. XPS spectra of Se (K) and Au (L) in AASP. M) The EPR method was used to determine the ability of 0–60 µg mL^−1^ AASP to scavenge hydroxyl radicals in vitro, with DMPO as the trapping agent. N) Ultrasound and photoacoustic imaging of AASP in vitro.

In the present study, L‐arg‐loaded selenium‐coated gold nanocages (AAS) were designed and synthesized. PCM modification yielded AASP, which targeted cardiomyocytes exhibited increased cellular uptake and improved photoacoustic imaging in vitro and in vivo. Se NPs first scavenged ROS, which prevented NO oxidation. Then, the L‐Arg that was loaded in the gold nanocores was catalyzed by nitric oxide synthase (eNOS) to produce NO, which in turn blocked ROS release by regulating the closing of the mitochondrial permeability transition pore (mPTP) (**Figure** **1**A). In vitro, AASP significantly blocked oxygen glucose deprivation/reoxygenation (OGD/R)‐induced H9C2 cell apoptosis by inhibiting oxidative damage and mitochondrial dysfunction. AASP administration in vivo effectively improved myocardial function and vascular remodeling, inhibited myocardial apoptosis and fibrosis, and ultimately attenuated MI/RI in rats by inhibiting oxidative damage and regulating NO signaling. Our findings confirmed the rational design of PCM‐modified selenium‐coated gold nanocages and showed their ability to enhance ROS inhibition and NO generation. These nanocages could be a highly efficient way to achieve targeted repair of heart tissue after MI/RI.

## 2 Results and Discussion

### 2.1 Synthesis and Characterization of Nanosystems

A graphical abstract that shows the route of synthesis and the mechanism underlying the effects of the L‐Arg‐loaded selenium‐gold nanocages is presented in Figure 1A. Our findings confirmed the rational design of the L‐Arg‐loaded selenium‐gold nanocages and showed that they had the potential to ameliorate myocardial ischemia/reperfusion injury by promoting nitric oxide production and maintaining mitochondrial function. Transmission electron microscopy (TEM) and Transmission electron microscopy (SEM) characterization results showed that hollow gold nanocages with diameters of 100 nm ± 12 nm and wall thicknesses of 7 ± 1.4 nm were prepared through the electrodisplacement reaction of HAuCl_4_ with Ag nanocubes. Layer‐by‐layer self‐assembly of cardiomyocyte‐targeting peptides and nanoselenium on the surfaces of the gold nanocages resulted in the preparation of AASP with a diameter of 150 ± 13 nm (Figure 1B–F; Figure S1A, Supporting Information). Elemental analysis confirmed that gold and selenium were uniformly distributed in the AASP (Figure 1G), with 11.2% and 6.7% Se and Au, respectively, while Ag nanocubes were successfully replaced by Au at only 2.5% of their weight (Figure S1C, Supporting Information). Like the TEM and SEM results, the successful surface modification of AASP with each component was accompanied by an increase in size and a change in positive and negative zeta potentials (Figure S1D,E, Supporting Information). The chemical structure of AASP was verified by UV‒vis‐NIR and FTIR spectroscopy techniques. As shown in Figure 1H, the characteristic absorption peaks at 900 and 275 nm in the AASP spectrum originate from the redshift of the Au nanocage absorption peaks and the intersection of the PCM and nanoselenium absorption peaks, respectively. As shown in the FTIR spectrum (Figure 1I) of AASP, the N‐H bending vibration absorption peak at 1558.20 cm^−1^ and the C = O stretching vibration absorption peak at 1641.13 cm^−1^ is derived from L‐arg and PCM. The crystal structure of AASP was further determined by XRD (Figure 1J), and the results showed the same diffraction peaks as those of trigonal Se (JCPDS No. 73–0465), cubic Au (JCPDS No. 04–0784) and oblique phase crystals Ag_3_AuSe_2_ (JCPDS No. 72–0392). The XPS analysis of Se 3d in the AASP showed binding energy peaks of 54.63, 54.93, 55.38, and 55.88 eV, which were attributed to Se^2‐^ 3d_5/2_, Se^0^ 3d_5/2_, Se^2‐^ 3d_3/2_ and Se^0^ 3d_3/2_, respectively. Au4f exhibited binding energy peaks of 83.78, 84.13, 87.48, and 87.88 eV, which were attributed to Au^0^4f_7/2_, Au^1+^4f_7/2_, Au^0^4f_5/2_ and Au^1+^4f_5/2_, respectively (Figure 1K,1L). The amounts of L‐arg and PCM in AASP were calculated with the regression equation established by UV spectrophotometry to be 119.2 and 21.5 µg mg^−1^, respectively (Figure S1F and G, Supporting Information). The electron paramagnetic resonance (EPR) results in Figure 1M confirm that AASP scavenges the ROS generated by the Fenton reaction in a concentration‐dependent manner, as evidenced by the reduced 1:2:2:1 EPR signal peak typical of 5,5‐dimethyl‐1‐pyrroline N‐oxide‐hydroxyl radical (DMPO‐^.^OH) adducts. Furthermore, loss of surface selenium nanoparticles and a reduction in particle size were observed in AASP after incubation with hydrogen peroxide (H_2_O_2_), and as a result, the L‐arg in AASP was continuously released, reaching equilibrium within 24 h (Figure S1H, Supporting Information).The results of the dimensional stability (Figure S1I, Supporting Information) and kinetic stability (Figure S1J, Supporting Information) studies showed that when incubated in pure water, PBS or DMEM supplemented with 10% fetal bovine serum (FBS), the nanoparticles still had good dispersibility for up to 72 h. In this study, gold nanocages served not only as drug release carriers but also as contrast agents to facilitate targeted photoacoustic imaging of the heart (Figure 1N). These results indicated that PCM‐modified L‐Arg‐loaded selenium‐gold nanocages had been successfully designed and synthesized.

### 2.2 AASP Attenuated OGD/R‐Induced H9C2 Cell Cytotoxicity

**Figure 2:**
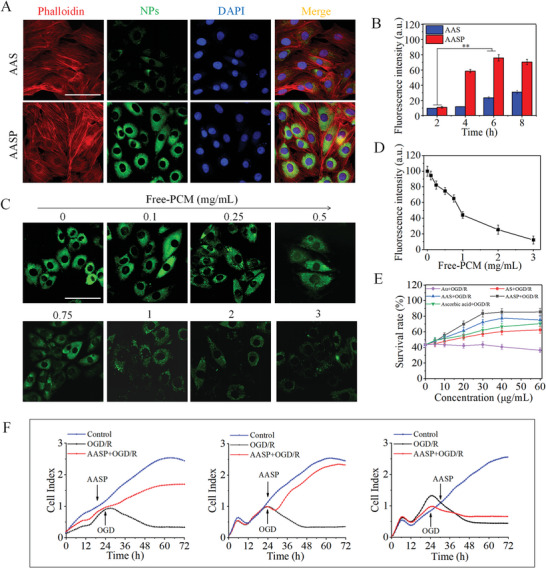
Cardiomyocyte‐specific uptake improved the cardiomyocyte survival rate. A) Colocalization of AASP, cytoskeleton and nucleus. H9C2 cells were contained to identify the cytoskeleton (red fluorescence), nucleus (blue fluorescence) and FITC‐labelled AASP (green fluorescence) and observed by confocal microscopy. Scale bar = 50 µm. B) Relative fluorescence intensity of H9C2 cells incubated with FITC‐labelled AAS and AASP for different times. C) Cardiomyocyte‐specific uptake of AASP. Free PCM (0.1, 0.25, 0.5, 0.75, 1.0, 2.0, and 3 mg mL^−1^) blocked AASP cell uptake. Scale bar = 50 µm. D) Quantitative analysis of cardiomyocyte‐specific uptake. E) AASP improved the survival rate of H9C2 cardiomyocytes after OGD/R treatment. F) Effects of pretreatment, cotreatment and posttreatment with AASP on H9C2 cell proliferation. All experiments were repeated at least three times and data are expressed as the mean ± SD, *p < 0.05, **p < 0.01.

First, the cellular uptake of AASP by H9C2 cells was assessed. As shown in **Figure** **2**A, H9C2 cells that were incubated with 30 µg mL^−1^ AASP for 8 h emitted bright green fluorescence in the cytoplasm, indicating that AASP effectively crossed the cell membrane and accumulated in the H9C2 cells. Real‐time fluorescence quantification further confirmed that the intracellular uptake of AASP occurred in a time‐dependent manner (Figure 2B). The intracellular fluorescence of AASP peaked at 6 h and was stronger than that of AAS, revealing that PCM modification facilitated the targeting of cardiomyocytes and enhanced the intracellular uptake of AASP. Figure 2C shows that free PCM prevented the uptake of FITC‐labelled AASP by cardiomyocytes, as evidenced by a decrease in green fluorescence. Quantitative fluorescence analysis further showed that free PCM concentration‐dependent manner prevented the accumulation of AASP in H9C2 cells (Figure 2D), which may result from free PCM competitively bound to tenascin X (PCM receptor). The effect of AASP on inhibiting OGD/R‐induced H9C2 cell cytotoxicity was subsequently evaluated. As shown in Figure 2E, OGD/R treatment significantly decreased the viability of H9C2 cells to 43.5%, suggesting that OGD/R caused obvious cytotoxicity. Cotreatment with AASP significantly inhibited OGD/R‐induced H9C2 cell cytotoxicity and exerted a better protective effect than Au nanocages, selenium‐coated gold nanocages (AS), ascorbic acid and AAS. Single‐cell Raman spectroscopy showed that AASP significantly improved the ability of cells to metabolize heavy water, which indirectly reflected the enhanced cell viability (Figure S2, Supporting Information). The changes in cell morphology further confirmed these protective effects (Figure S3, Supporting Information). Notably, Au nanocages alone did not exhibit effective treatment of OGD/R‐induced cytotoxicity. Live/dead cell staining clearly demonstrated that the AASP significantly inhibited OGD/R‐induced H9C2 cell death, as indicated by decreased red fluorescence (Figure S4A and B, Supporting Information). Pretreatment, cotreatment and posttreatment with AASP significantly attenuated OGD/R‐induced H9C2 cell cytotoxicity (Figure 2F), and real‐time monitoring also suggested that cotreatment with AASP and OGD/R was the optimal design. Moreover, the flow cytometry results further showed that AASP cotreatment significantly inhibited OGD/R‐induced H9C2 cell apoptosis, and statistical analysis of the proportions of apoptotic cells further confirmed this conclusion (Figure S4C and D, Supporting Information). Taken together, these results suggested that PCM modification facilitated the targeting of cardiomyocytes and enhanced the intracellular uptake of AASP, which ultimately attenuated OGD/R‐induced H9C2 cell cytotoxicity and apoptosis.

### 2.3 AASP Promoted NO Production

**Figure 3:**
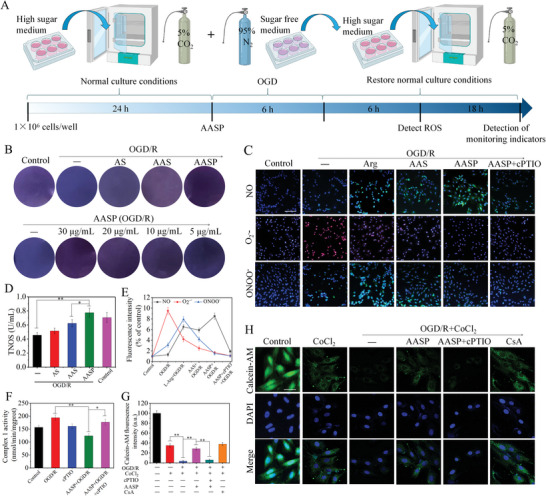
AASP promoted NO production. A) Schematic diagram of OGD/R model building, dosing, and detection times. B) TNOS and L‐Arg catalyzed the production of NO, which combined with nucleophiles to generate colored compounds to measure the TNOS viability of cells after different treatments. Fluorescence imaging (C) and fluorescence intensity statistics (E) of NO (DAF‐FMDA), O_2_^.−^ (DHE) and ONOO^−^ (HPF) produced in cells after different treatments, and cPTIO was used as a negative control to scavenge NO. Scale bar = 50 µm. D) Effect of AS, AAS and AASP (30 µg mL^−1^) on TNOS activity in OGD/R‐treated cells. F) Effects of NO on mitochondrial complex I activity in cardiomyocytes. Fluorescence intensity statistics (G) and fluorescence imaging (H) of mPTP opening levels, with CsA as the positive control. Scale bar = 20 µm. All experiments were repeated at least three times and data are expressed as the mean ± SD, *p < 0.05, **p < 0.01.

A timeline of the in vitro experimental design is presented in **Figure** **3**A. L‐Arg is a substrate that can be catalyzed by total nitric oxide synthase (TNOS) to produce NO. Increasing numbers of studies have confirmed that NO, which is an important messenger molecule, plays a key role in regulating cardiovascular function by affecting mitochondrial function and energy metabolism of the myocardium.^[^ ^17^ ^]^ Herein, we first examined TNOS activity using a TNOS kit. TNOS catalyzes the reaction between L‐Arg and molecular oxygen to produce NO, and NO reacts with nucleophiles to produce colored compounds. The absorbance was measured at 530 nm, and the TNOS activity could be calculated according to the magnitude of absorbance. As shown in Figure 3B, H9C2 cells exposed to OGD/R treatment reacted with nucleophiles to produce blue compounds, indicating decreased TNOS enzyme activity (Figure 3D). However, AASP cotreatment significantly maintained the TNOS activity, as shown by the red compounds produced by H9C2 cells reacting with nucleophilic substances. Second, NO production was measured with a Diaminofluorescein‐FM diacetate (DAF‐FMDA) probe. As shown in Figure 3C, AASP cotreatment significantly promoted NO production in H9C2 cells, as demonstrated by enhanced green fluorescence. However, NO rapidly reacts with excessive levels of superoxide anion (O2^.−^) in myocardial tissue subjected to MI/RI, and this reaction generates peroxynitrite anion (ONOO^−^), which further damages the tissues.^[^ ^14b^ ^]^ OGD/R‐treated cardiomyocytes produced large amounts of O2^.−^, and excess ONOO^−^ was detected after L‐Arg treatment, resulting in decreased cell viability (Figure S6A, Supporting Information). AASP cotreatment significantly reduced the production of O_2_^.−^ and ONOO^−^ in H9C2 cells and significantly increased the cell survival rate after OGD/R treatment (Figure 3E). Thus, protection from NO oxidation is a critical step in NO treatment. These results clearly revealed that AASP cotreatment restored TNOS activity and promoted NO generation, and this treatment exerted stronger effects than AAS and Arg. To further confirm the significance of NO, an NO inhibitor (cPTIO) was used, and the results showed that inhibition of NO by cPTIO significantly attenuated the protective effects of AASP on NO production and H9C2 cell viability.

The activation of complex I in mitochondria is positively correlated with ROS generation.^[^ ^18^ ^]^ To investigate the causal relationship between NO generation and ROS inhibition, complex I activity was examined by an NO inhibitor (cPTIO). As shown in Figure 3F, OGD/R treatment resulted in obvious complex I activation, which indirectly indicated ROS generation. AASP cotreatment significantly inhibited complex I activation in OGD/R‐treated H9C2 cells. The addition of cPTIO significantly blocked the AASP‐induced inhibition of complex I activation. These results revealed that AASP promoted NO production, which in turn blocked ROS generation.

The opening of the mitochondrial permeability transition pore (mPTP) can lead to ROS release.^[^ ^19^ ^]^ To elucidate the underlying mechanism by which NO blocked ROS generation, mPTP opening was evaluated by calcein‐AM and CoCl_2_. Calcein‐AM can cross normal cell membranes and mitochondrial membranes and emits green fluorescence. CoCl_2_ can cross the normal cell membrane and quench the green fluorescence of Calcein‐AM in the cytoplasm but cannot cross the normal mitochondrial membrane, so the green fluorescence in the mitochondria is retained. However, when mPTP is open, CoCl_2_ enters the mitochondria and quenches the green fluorescence of Calcein‐AM in the mitochondria. As shown in Figure 3H, the mitochondria of control H9C2 cells still emitted distinct green fluorescence after CoCl_2_ treatment. However, the mitochondrial fluorescence of H9C2 cells exposed to OGD/R treatment was significantly quenched by CoCl_2_. In contrast, H9C2 cells that were cotreated with AASP and OGD/R still emitted significant green fluorescence; this result revealed that AASP cotreatment promoted NO generation, and NO generation in turn regulated mPTP closing and blocked ROS release from the mitochondria. The addition of an NO inhibitor (cPTIO) resulted in a significant decrease in cell fluorescence intensity, further confirming this conclusion (Figure 3G). Taken together, these results suggested that AASP promoted the production of NO, which blocked ROS release from the mitochondria by regulating the closing of the mPTP.

### 2.4 AASP Maintained Mitochondrial Function in H9C2 Cells

**Figure 4:**
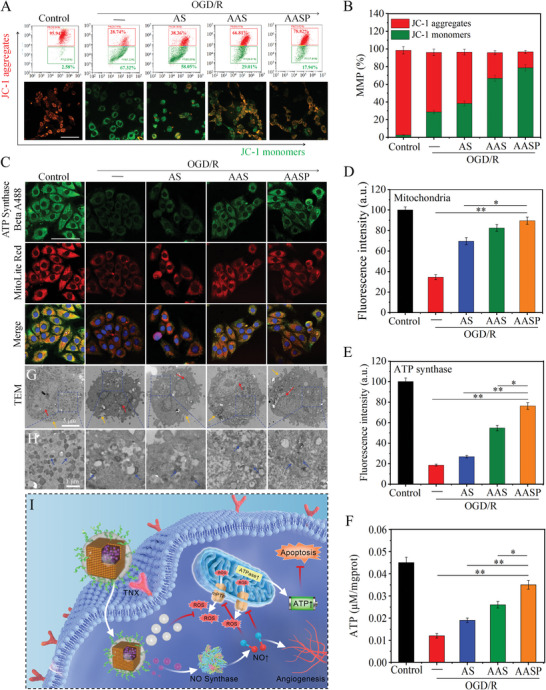
AASP maintained mitochondrial function. A) AASP maintained H9C2 cell MMP after OGD/R treatment. H9C2 cells were labelled with a JC‐1 probe and analyzed by flow cytometry and fluorescence microscopy. Scale bar = 50 µm. B) Statistical analysis of the MMP. C) AASP restored ATP synthase activity and maintained mitochondrial integrity. Colocalization of ATP synthase, mitochondria, and nuclei by laser confocal microscopy. Scale bar = 50 µm. Mitochondria (D) and ATP synthase (E) were fluorescently labelled by MitoLite Red and ATP synthase βA488, respectively, and the relative fluorescence intensity was recorded. F) Luciferase was used to measure ATP levels. G) The ultrathin sections of cells were observed by TEM, where the red and yellow arrows point to the nucleus and the cell membrane, respectively. H) Magnified TEM images of ultrathin sections, where the blue arrows indicate the mitochondria inside the cell. I) AASP improves apoptosis in in vitro oxygen‐glucose deprivation models by promoting nitric oxide production and maintaining mitochondrial function. All experiments were repeated at least three times, and data are expressed as the mean ± SD, *p < 0.05, **p < 0.01.

The mitochondrial membrane potential (MMP) was first evaluated in H9C2 cells. **Figure** **4**A shows that AASP cotreatment effectively improved the MMP in OGD/R‐treated H9C2 cells, as evidenced by the shift in fluorescence from green to red. The flow cytometry results and quantitative analysis both further confirmed this conclusion (Figure 4A, B). ATP synthase in mitochondria can catalyze and synthesize ATP and plays a key role in regulating cellular metabolism. The dysfunction of ATP synthase is accepted as one of the important mechanisms underlying mitochondrial dysfunction and myocardial apoptosis.^[^ ^20^ ^]^ Herein, ATP synthase activity in H9C2 cells was assayed by fluorescence containing for ATP synthase and mitochondria. As shown in Figure 4C, OGD/R treatment significantly decreased ATP synthase activity and mitochondrial number, as demonstrated by the decreased green and red fluorescence, respectively. Quantification of the fluorescent signals further confirmed this result (Figure 4D,E). TEM analysis of the cellular ultrastructure showed that OGD/R treatment caused obvious damage to the cell membrane, decreased mitochondrial numbers, and disrupted the nucleus (Figure 4G,H). However, AASP cotreatment significantly improved the cellular ultrastructure, restored ATP synthase activity and mitochondrial number, and increased the ATP content in OGD/R‐induced H9C2 cells (Figure 4F). AASP cotreatment exerted better protective effects than AS, ascorbic acid and AAS.

Mitochondrial dysfunction may lead to mPTP opening and cause the release of ROS, which could cause oxidative damage in H9C2 cells. An increasing number of studies have shown that OGD/R can induce ROS overproduction and cause oxidative damage to cardiomyocytes.^[^ ^21^ ^]^ Herein, 2,7‐dichlorodihydrofluorescein diacetate (DCFH‐DA) was used to investigate the oxidative status of H9C2 cells. As shown in Figure S5C (Supporting Information), OGD/R treatment caused significant ROS generation in a time‐dependent manner, and ROS levels peaked at 6 h. However, AASP cotreatment significantly reduced OGD/R‐induced ROS generation in a time‐dependent manner, and AASP exerted better protective effects than AS, ascorbic acid and AAS. The decreased green fluorescence (Figure S5A, Supporting Information), fluorescent quantification results (Figure S5B, Supporting Information), and flow cytometry results (Figure S5D, Supporting Information) all confirmed this conclusion. Excessive levels of ROS can oxidize lipids in biological membranes to form lipid peroxidation products (MDA), resulting in changes in cell structure and function. Hence, the superoxide dismutase (SOD) and glutathione peroxidase (GSH‐Px) activities and MDA content were measured. As shown in Figure S5E–G (Supporting Information), AASP cotreatment significantly increased the activities of SOD and GSH‐Px and decreased the content of MDA in OGD/R‐induced H9C2 cells. We speculated that excessive ROS production may exceed its own ability to scavenge ROS, increasing the depletion of SOD and GSH‐Px. However, selenium represented an active site of many selenium‐containing enzymes, and the selenium contained in AASP could effectively inhibit ROS accumulation, ultimately reducing the depletion of SOD and GSH‐Px. In summary, AASP exerted increased protective effects against OGD/R‐induced oxidative damage by depleting reactive oxygen species and protecting NO production, thereby maintaining mitochondrial function in H9C2 cells by mechanisms such as regulating mPTP shutdown, promoting ATP synthesis and maintaining mitochondrial number (Figure 4I).

### 2.5 AASP Improved Myocardial Function in MI/RI Model Rats

**Figure 5:**
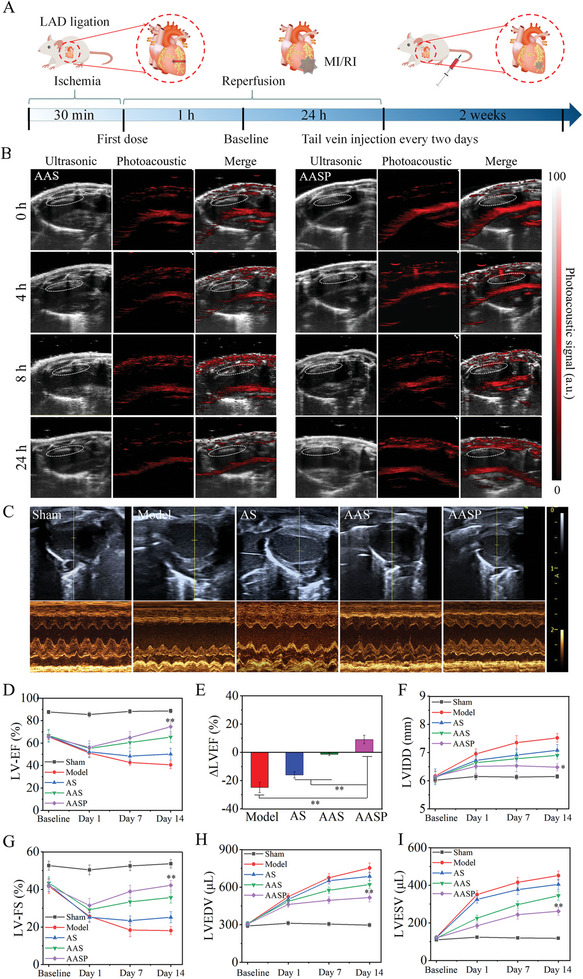
Cardiomyocyte‐specific photoacoustic imaging and improved myocardial function in MI/RI model rats. A) Schematic diagram of the animal MI/RI model and the time points. B) Cardiomyocyte‐specific photoacoustic imaging. AASP was injected via the tail vein and imaged in the MI/RI rat left ventricle by ultrasound, and photoacoustic signals were measured at 0, 4, 8, and 24 h. C) Echocardiographic imaging was performed on postoperative day 14. Indexes of cardiac function, such as LV‐EF (D), LVEF (E), LV‐FS (F), LVIDD (G), LVEDV (H) and LVESV (I), were evaluated by echocardiography at different time points after the operation. The data are expressed as the mean ± SD, *n* = 10 animals per group, *p < 0.05, **p < 0.01.

A timeline of the in vivo experimental design is presented in **Figure** **5**A. The use of advanced nanoprobes with enhanced photoacoustic properties for noninvasive in vivo visual imaging of vulnerable atherosclerosis was previously described.^[^ ^22^ ^]^ MI/RI model rats were injected with AASP via the caudal vein, and the cardiomyocyte‐targeting property of AASP in vivo was monitored in real time. Figure 5B shows that the photoacoustic (PA) signal was visibly detected in the anterior wall of the left ventricle at 4 h, and the PA signal of AASP was 3 times stronger than that of AAS (Figure S6B). The PA signal intensity showed a significant decline, indicating that AASP can be quickly metabolized in vivo. These results indicated that after PCM modification, AASP improved cardiomyocyte‐specific photoacoustic imaging in vivo.

Myocardial reperfusion therapy, which is the most effective way to restore blood and oxygen supply to ischemic heart tissues, may in turn cause lethal tissue damage to the heart.^[^ ^23^ ^]^ The identification of new strategies to mitigate MI/RI remains a great challenge in the clinic. Therefore, myocardial function in MI/RI model rats after the administration of AASP was evaluated. Lactate dehydrogenase (LDH) and creatine kinase‐isokinase MB (CK‐MB) are important indicators of MI/RI, and they were first examined.^[^ ^24^ ^]^ As shown in Figure S6C and D (Supporting Information), the MI/RI model rats showed significant increases in LDH and CK‐MB levels, indicating the successful establishment of the MI/RI model. Echocardiographic assessment revealed that MI/RI resulted in significant left ventricular systolic dysfunction (Figure 5C). Quantitative analysis of the indicators (Figure 5D–I), such as left ventricular ejection fraction (LV‐EF), left ventricular fraction shortening (LV‐FS), left ventricular internal diameter at end‐diastole (LVIDD), left ventricular end‐diastolic volume (LVEDV), and left ventricular end‐systolic volume (LVESV), further confirmed myocardial dysfunction in the MI/RI model rats. However, AASP cotreatment significantly decreased LDH and CK‐MB levels and normalized LV‐EF, LV‐FS, LVIDD, LVEDV and LVEVS. The protective effects of AASP were much better than those of AS and AAS. Taken together, these results indicated that AASP improved cardiomyocyte‐specific photoacoustic imaging and improved myocardial function in MI/RI model rats.

### 2.6 AASP Inhibited Oxidative Damage and Myocardial Apoptosis in MI/RI Model Rats

The mechanism underlying AASP‐mediated protection against MI/RI in rats was further explored by histological analysis. As shown in Figure S7A (Supporting Information), H&E staining showed obvious inflammatory cell infiltration in the ventriculus sinister of the MI/RI model rats. Active caspase‐3 staining (Figure S7B and E, Supporting Information) and TUNEL staining (Figure S7C and F, Supporting Information) revealed significant myocardial apoptosis in the ventriculus sinister of the MI/RI model rats. Moreover, the induction of oxidative damage was also observed in the MI/RI model rats, as demonstrated by the decreased activity of the antioxidant enzymes SOD (Figure S7H, Supporting Information) and GSH‐Px (Figure S7I, Supporting Information) and the increased MDA content (Figure S7J, Supporting Information). However, AASP administration in vivo dramatically inhibited inflammatory cell infiltration, myocardial apoptosis, and oxidative damage in MI/RI model rats.

**Figure 6:**
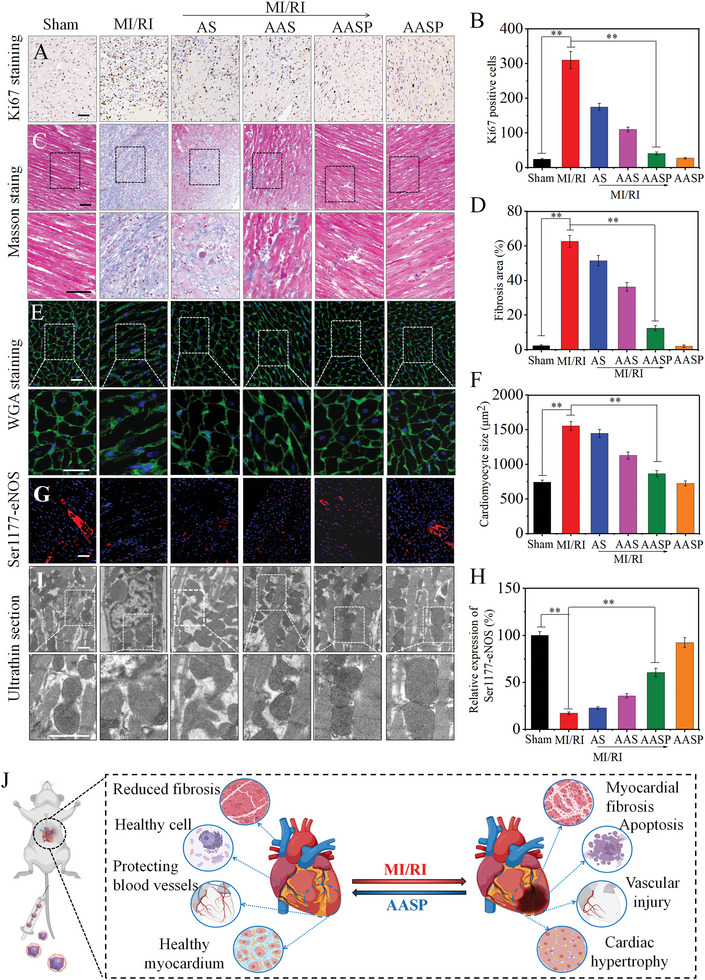
AASP protected eNOS activity and improved cardiomyocyte proliferation and vascular remodeling in MI/RI model rats. A) Ki67‐labelled proliferating cells in the MI/RI regions of rats and B) quantitative assessment of Ki67‐positive cells. Scale bar = 50 µm. C) The degree of myocardial fibrosis in the MI/RI region of each treatment group was analyzed by Masson staining, and D) the area of myocardial fibrosis was assessed (the scale bar in both the pre‐ and postmagnification images is 50 µm). E) WGA staining shows cardiomyocyte hypertrophy, characterized by increased cross‐sectional area (the scale bar in both the pre‐ and postmagnification images is 50 µm). F) Quantification of the cross‐sectional area of cardiomyocytes. G) Immunofluorescence analysis of the Ser1177‐eNOS levels in the MI/RI regions of rats in each treatment group and H) quantitative assessment of the Ser1177‐eNOS levels. Scale bar = 50 µm. I) The ultrastructure of myocardial mitochondria in the MI/RI region of the rats in each treatment group was observed by TEM. Scale bar = 1 µm. The data are expressed as the mean ± SD, *n* = 10 animals per group, *p < 0.05, **p < 0.01. J) Schematic representation of cardiac changes in rats before and after AASP treatment of MI/RI.

Protection of the vascular system facilitates myocardial regeneration and repair after MI/RI. CD34 staining demonstrated that AASP administration in vivo ameliorated vascular injury in the MI/RI model rats (Figure S7D and G, Supporting Information). Ki‐67 staining revealed that AASP administration in vivo inhibited the abnormal proliferation of cardiac fibroblasts in the MI/RI model rats (**Figure** **6**A,B), which may be responsible for the reduction in myocardial fibrosis. Masson staining indicated that the MI/RI model rats had significant myocardial fibrosis, as evidenced by the increased collagen fiber staining (Figure 6C,D). An increased cross‐sectional area of cardiomyocytes is characteristic of dilated cardiomyopathy and ventricular systolic dysfunction. WGA staining clearly indicated that AASP administration in vivo effectively blocked the expansion of cardiomyocytes (Figure 6E,F). Staining for Ser1177‐eNOS showed that AASP administration in vivo significantly maintained the activity of Ser1177‐eNOS (Figure 6G,H), which could induce NO production and thus maintain mitochondrial function and number. TEM observation showed that AASP administration in vivo effectively attenuated myocardial fiber rupture and mitochondrial damage and increased mitochondrial numbers (Figure 6I). In all these experiments, AASP exerted better effects than AS and AAS. Taken together, these results suggested that AASP administered in vivo effectively improved cardiac fibroblast proliferation and vascular injury, reduced cardiomyocyte apoptosis and inhibited the expansion of cardiomyocytes in MI/RI model rats (Figure 6J).

### 2.7 Transcriptomic Analysis of Molecular Mechanism

**Figure 7:**
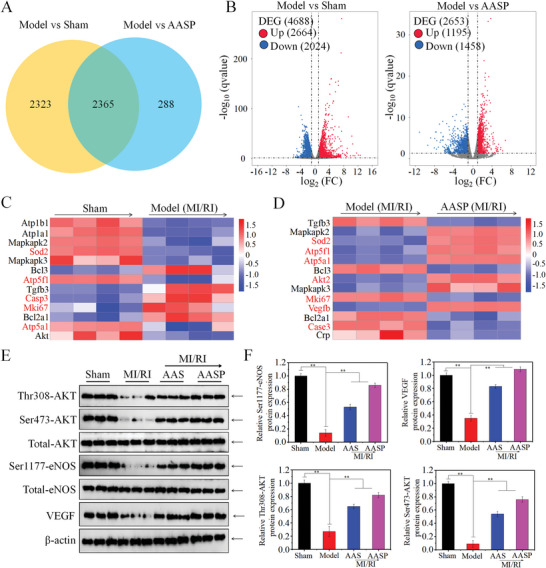
Transcriptomic analysis of the molecular mechanism. Venn diagram A) and volcano plot B) of differentially expressed genes between the model group and sham operation group, and the model group and AASP treatment group. C) Heatmap analysis of differentially expressed genes in the myocardium of the sham operation group and model group. D) Heatmap analysis of differentially expressed genes in the myocardium of MI/RI rats after AASP treatment versus the myocardium of model rats. The levels of AKT/eNOS pathway‐related proteins E) and quantitative analysis F) in the MI/RI region were determined by Western blotting. All experiments were repeated at least three times and data are expressed as the mean ± SD, *p < 0.05, **p < 0.01.

To investigate the mechanism underlying the protective effects of AASP against MI/RI in rats, transcriptomic analysis of the left ventricle from MI/RI model rats was conducted to measure the differences in mRNA expression across the entire transcriptomic analysis. As shown in the Venn diagram (**Figure** **7**A) and the volcano diagram (Figure 7B), there were 4688 differentially expressed genes between the sham group and model group. The model group and the AASP‐treated group also had 2653 differentially expressed genes. Gene Ontology (GO) enrichment analysis of biological process (BP), cellular component (CC) and molecular function (MF) showed that ATPase activity, ATP metabolic processes, angiogenesis, myocardial contraction, mitochondrial respiratory chain, oxidoreductase activity, and fatty acid oxidation were all enriched after AASP administration in vivo (Figure S8A, Supporting Information). Kyoto Encyclopedia of Genes and Genomes (KEGG) pathway analysis showed that the VEGF signaling pathway, PI3K‐Akt signaling pathway, cAMP signaling pathway, selenium compound metabolism, glutathione metabolism, arginine and proline metabolism pathways were enriched after AASP administration in vivo (Figure S8B, Supporting Information). Heatmap analysis showed that the expression of the Sod2, Atp5f1 and Atp5a1 genes was downregulated, while the expression of the Mki67 and Casp3 genes was upregulated in the myocardium of rats in the MI/RI model group compared to the sham‐operated group (Figure 7C). In contrast, the heatmap specifically revealed that AASP administration in vivo significantly upregulated Sod2, Atp5f1, Atp5a1, Vegfb, and Akt expression and downregulated Mki67 and Casp3 expression in the MI/RI model rats (Figure 7D). The protein expression levels of Thr308‐AKT, Ser473‐AKT, Ser1177‐eNOS and VEGF were measured by Western blotting, and the results further confirmed this conclusion (Figure 7E,F). Taken together, these results suggested that AASP administration in vivo significantly attenuated MI/RI in rats, which was accompanied by improved ATP synthesis, angiogenesis, and oxidoreductase metabolism.

### 2.8 Evaluation of AASP Safety

The distribution and biocompatibility of nanodrugs in vivo are both important factors in drug design and development. Herein, the biodistribution and biocompatibility of AASP were evaluated in rats. As shown in Figure S9 (Supporting Information), AASP mainly accumulated in the heart at 4 h and then accumulated in the other organs. The liver was the main organ where AASP was metabolized, and this metabolism peaked at 12 h (Figure S9A and B, Supporting Information). The contents of Se (Figure S9C, Supporting Information) and Au (Figure S9D, Supporting Information) in the main organs in vivo gradually decreased in a time‐dependent manner, and almost no retention was observed at 21 d, indicating that AASP can be quickly metabolized. Figure S10 (Supporting Information) shows that AASP administration in vivo caused no significant changes in hemolytic behavior (Figure S10A, Supporting Information) or rat body weight (Figure S10B, Supporting Information). Analysis of blood biochemical indicators showed that AASP exerted no significant effects on alanine aminotransferase (ALT), aspartate aminotransferase (AST), blood urea nitrogen (BUN) or creatinine (CRE) (Figure S10C–F, Supporting Information). Histopathological analysis by H&E staining suggested that AASP administration in vivo caused no significant histopathological changes in the heart, liver, spleen, lung, kidney, or brain (Figure S11, Supporting Information). Taken together, these results indicated that AASP showed good safety and biocompatibility in vivo.

## 3 Conclusion

In summary, we reported an L‐arg‐loaded selenium‐coated gold nanocage with myocardial targeting properties (AASP). AASP after PCM modification achieved cardiomyocytes‐targeted recognition and enhanced cellular uptake. Se NPs first scavenged ROS in cardiomyocytes of the OGD/R model, provided a suitable environment for the release of NO and prevented its further oxidation to peroxynitrite anions. Then, the L‐arg loaded in gold nanocages was released and NO was produced catalyzed by nitric oxide synthase, which in turn blocked ROS release by regulating the closing of the mPTP. Thus, AASP prevented cardiomyocyte apoptosis in vitro by scavenged ROS and produced NO to maintain mitochondrial function. Further, AASP effectively improves myocardial function in vivo by maintaining mitochondrial function and regulating NO signaling, inhibiting cardiomyocyte apoptosis and fibrosis, and ultimately decreasing MI/RI in rats. The AASP nanosystem has shown great potential for targeted delivery and synergistic therapy in the treatment of MI/RI.

## 4 Experimental Section

### Materials

Chloroauric acid (HAuCl_4_·3H_2_O, 99.9%), silver nitrate (AgNO_3_, 99%), sodium selenite (Na_2_SeO_3_, 98%), Dimethyl sulfoxide (DMSO), DMPO (98%), polyvinylpyrrolidone K30 (PVP‐K30), cetyltrimethylammonium bromide (CTAB, 98%), and L‐Arg (98%) were purchased from Sigma‒Aldrich (Mainland China). Fluorescein isothiocyanate n‐hydrox ysuccinimide (FITC‐NHS, 90%), 4′,6‐diamidino‐2‐phenylindole (DAPI, 95%), rhodamine phalloidin (95%), DCFH‐DA (95%), DAF‐FMDA (95%), dihydroethidium (DHE, 95%), hydroxyphenyl fluorescein (HPF, 95%) were purchased from Thermo Fisher Scientific (Mainland China). PCM (WLSEAGPVVTVRALRGTGSW) was synthesized by Shanghai Bioengineering Co. The purity of the synthesized PCM was determined to be higher than 95% by mass spectrometry and high‐performance liquid chromatography detection.

### Synthesis of Gold Nanoparticles (Au NPs)

The Au NPs were synthesized by a substitution reaction between Ag NPs and HAuCl_4_·3H_2_O.^[^ ^25^ ^]^ Briefly, 650 mg of PVP‐K30, 340 mg of AgNO_3_ and 10 mg of NaCl were dissolved in 7 mL of ethylene glycol, and then, 2 mL of glycerol was added and allowed to react 150 °C for 2 h to obtain Ag NP colloids. Two hundred microlitres of Ag NP colloidal solution was dispersed in 20 mL of deionized water, heated, and boiled for 10 min. Then, 5 mL of HAuCl_4_ (1 mm) was slowly added, and reflux was continued for 20 min under ultrasonication. The AgCl that was generated by the displacement reaction was dissolved and removed with a saturated NaCl solution, and the Au NPs were obtained by centrifugation (8000 g, 15 min).

### Synthesis of L‐Arg@Au@Se@PCM Nanoparticles (AASP NPs)

Eight milliliters of Au NP (0.1 mg mL^−1^) solution and 2 mL of L‐Arg (20 mg mL^−1^) solution were mixed and stirred for 2 h and centrifuged (8000 g, 15 min) to obtain L‐arg‐modified gold nanocages. Five hundred microliters of cetyltrimethylammonium bromide (CTAB, 10 mg mL^−1^) solution and 300 µL of Na_2_SeO_3_ (17.3 mg mL^−1^) solution were mixed with the redispersed L‐arg‐modified gold nanocage solution (3 mL), and then, 1.2 mL of ascorbic acid solution (17.6 mg mL^−1^) was added in a dropwise manner. After stirring for 30 min, the color of the solution became and remained tan, and this color remained. PCM at a final concentration of 5 mg mL^−1^ was added to the reaction system and incubated overnight at 4 °C; then, the system was centrifuged (8000 g, 15 min) and washed to remove unreacted substances. FITC‐labelled nanoparticles were prepared according to a previously reported method with slight modifications.^[^ ^26^ ^]^ Briefly, 2 mL of AAS or AASP solution (4 mg mL^−1^, pure water) was mixed with 0.1 mL of FITC‐NHS solution (19 mg mL^−1^, DMSO) and stirred at 4 °C overnight. The product was collected by centrifugation (8000 g, 15 min) and purified by washing three times with deionized water for purification, followed by lyophilization.

### Physicochemical Characterization

High‐definition transmission electron microscopy (TEM, HT7700, Hitachi, Japan), scanning electron microscopy (SEM, S‐4800, Hitachi, Japan) and energy dispersive X‐ray spectroscopy (EDS, X‐Max‐N150, Oxford, UK) were used for morphological observation and elemental analysis of the nanoparticles. Nanoparticle size and surface zeta potential were measured by dynamic light scattering (DLS) using a Nano‐ZS instrument (Malvern Instruments Limited, UK). Ultraviolet‐near infrared (UV‒vis‐NIR) absorption spectra were detected by a UV‒vis‐NIR spectrophotometer (UV‐2550, Shimadzu, Japan). Fourier transform infrared (FTIR) spectroscopy was performed with an FTIR spectrometer (Nicoletteis50; Thermo Fisher Scientific, USA) in the wavelength range of 4000–500 cm^−1^. X‐ray photoelectron spectroscopy (XPS, EscaLab Xi^+^, Thermo Fisher Scientific, USA) was used to analyze the elemental composition and valence of the nanoparticles. X‐ray diffraction (XRD, D/max‐2400, Tokyo, Japan) was used to analyze the crystal structure of the nanospheres. EPR spectra were recorded using a Bruker EMX EPR spectrometer with DMPO as the reactive oxygen trapping agent. In vivo photoacoustic (PA) imaging was conducted with a PA system (Endra Nexus 128, USA).

### Cell Culture

H9C2 cells (rat cardiomyocytes) were purchased from the Cell Resource Center, Institute of Basic Medical Sciences, Chinese Academy of Medical Sciences. The cells were cultured in high‐glucose Dulbecco's modified Eagle's medium (DMEM) supplemented with 10% FBS, 100 IU mL^−1^ penicillin and streptomycin in a carbon dioxide incubator (37 °C and 5% CO_2_). An oxygen‐glucose deprivation/reoxygenation (OGD/R) model was established with glucose‐free DMEM, and the cells were incubated in 95% N_2_ and 5% CO_2_ for 6 h. Then, the cells were cultured under normal conditions for 24 h to allow reoxygenation.

### Real‐Time Cell Analysis

A real‐time cell electronic sensor system (xCELLigence RTCA‐S16, ACEA Bioscience, USA) was used to measure cell proliferation. Briefly, H9C2 cells were cultured in e‐plates at a density of 5×10^3^ cells per well for 24 h at 37 °C. Then, AASP NPs (30 µg mL^−1^) were added to the e‐plates, and the cells were exposed to OGD/R. Real‐time and dynamic cell proliferation was measured within 72 h. Similarly, AASP NPs were added to the cells 6 h before OGD/R treatment or 6 h after OGD/R treatment, and real‐time and dynamic cell proliferation was measured within 72 h.

### Cellular Uptake of L‐Arg@Au@Se NPs and AASP NPs

H9C2 cells (1×10^6^ cells per well) were seeded in 6‐well plates and cultured for 24 h at 37 °C. FITC‐labelled nanoparticles (30 µg mL^−1^) were added to the 6‐well plates to observe the changes in the cell fluorescence intensity within 8 h. Similarly, the cells were preblocked with free PCM (0, 0.1, 0.25, 0.5, 0.75, 1.0, 2.0, and 3.0 mg mL^−1^) for 24 h, and then, 30 µg mL^−1^ FITC‐labelled nanoparticles were added to the 6‐well plates to observe the intracellular fluorescence intensity at the 6 h time point. A multifunctional microplate reader (Tecan Infinite 200Pro, Austria, Switzerland) was used to measure the fluorescence intensity of FITC (excitation and emission wavelengths of 495 and 519 nm, respectively), and the cellular uptake was expressed as the percentage of adsorbed nanoparticles. The distribution of the nanoparticles in the cell was determined by colocalizing the nanoparticles with the cytoskeleton (rhodamine phalloidin, excitation wavelength and emission wavelength of 540 and 570 nm, respectively) and cell nucleus (DAPI, excitation wavelength and emission wavelength of 358 and 461 nm, respectively), and the results were recorded by confocal laser microscopy (TCS SP8 X, Leica, Germany).

### Cell Viability Assay

H9C2 cells (5×10^3^ cells per well) were seeded in 96‐well plates and cultured for 24 h at 37 °C. Nanoparticles (0, 10, 20, 30, 40, and 50 µg mL^−1^) were added to 96‐well plates, and then, the cells were subjected to OGD/R treatment. A Cell Counting Kit‐8 (ab228554, Abcam) was used to determine cell viability. Briefly, H9C2 cells (1×10^6^ cells well^−1^) were seeded into 6‐well plates and incubated for 24 h at 37 °C. Nanoparticles (30 µg mL^−1^) were added to 6‐well plates, and then, the cells were subjected to OGD/R treatment. The cells were stained for 30 min according to the LIVE/DEAD kit (L3224, Thermo Fisher Scientific). The cells were imaged by fluorescence microscopy.

### Single‐Cell Raman Spectroscopy was used to Assess the General Metabolic Activity of the Cells

H9C2 cells (5×10^3^ cells per well) were seeded in 96‐well plates and cultured for 24 h at 37 °C. NPs (30 µg mL^−1^) were added to the 96‐well plates, and the cells were subjected to OGD/R treatment (30% heavy water (v/v)‐containing medium). Approximately 20 single cells from each group were randomly sampled for Raman spectroscopy measurements after OGD/R treatment. The C‐D ratio, which was an index that was used to quantify the substitution of D atoms in C─H bonds and the metabolic states of single cells, was calculated by dividing the integrated area intensity of the C‐D band (2040–2300 cm^−1^) by the sum of the C‐D band and C–H band (2800–3100 cm^−1^) via Ramanome Explorer (RamEX, Qingdao Single Cell Biotech, China).

### Flow Cytometry Analysis

The ratio of the decrease in the cell mitochondrial membrane potential and cell apoptosis after nanoparticle treatment was analysed by flow cytometry. H9C2 cells (1×10^6^ cells well^−1^) were seeded in 6‐well plates and cultured for 24 h at 37 °C. Nanoparticles (30 µg mL^−1^) were added to 6‐well plates, and the cells were subjected to OGD/R treatment. After treatment, all the cells were collected, and the cells were stained according to the instructions of the apoptosis detection kit (331 200, Thermo Fisher Scientific) and MMP detection kit (V35116, Thermo Fisher Scientific). Then, the stained cells were analyzed by flow cytometry (Cytoflex, Beckman, CA, USA).

### Measurement of ROS Generation

To confirm the production of intracellular ROS, H9C2 cells were seeded in 6‐well plates at a density of 1×10^6^ cells per well and cultured for 24 h. Nanoparticles (30 µg mL^−1^) were added to the 6‐well plates, and the cells were subjected to OGD/R treatment. After 6 h of reoxygenation, the medium was removed, and the cells were gently washed with PBS three times. Then, 10 µm DCFH‐DA (excitation wavelength and emission wavelength of 495 and 520 nm, respectively), DHE (excitation wavelength and emission wavelength of 518 and 606 nm, respectively) and HPF (excitation wavelength and emission wavelength of 490 and 515 nm, respectively) were added to each well separately and incubated at 37 °C for 45 min. Finally, 0.3 µm DAPI (excitation wavelength and emission wavelength of 358 and 460 nm, respectively) was added to stain the cell nuclei for 5 min. Photographic recordings were made by inverted fluorescence microscopy (Nikon, Ti‐E), and the fluorescence intensity of the cells was measured to verify the production of intracellular ROS.

### Measurement of SOD and GSH‐PX Activities and MDA Content in H9C2 Cells

Superoxide dismutase (SOD) activity was measured by xanthine oxidase assay. Briefly, after OGD/R and nanoparticle treatment, H9C2 cells were collected and fragmented by ultrasonic fragmentation. The supernatants were collected by centrifugation, and the protein concentration was quantified. Then, the absorbance was measured at 450 nm with a multifunctional microplate reader according to the SOD activity assay kit (19 160, Sigma‒Aldrich) procedure. The activity of GSH‐Px was measured by a chemical colorimetric method. After treatment as described above, the absorbance was measured at 412 nm with a multifunctional microplate reader according to the GSH‐Px kit (CGP1, Sigma‒Aldrich). Lipid peroxidation activity was measured by a thiobarbituric acid assay. After treatment as described above, the absorbance was measured at 532 nm with a multifunctional microplate reader according to the MDA kit (MAK085, Sigma‒Aldrich).

### Production of Nitric Oxide (NO)

H9C2 cells (5×10^3^ cells well^−1^) were seeded in 96‐well plates and cultured at 37 °C for 24 h. Nanoparticles (0–30 µg mL^−1^) were added to 96‐well plates, and the cells were subjected to OGD/R treatment. The cells were collected and lysed at 6 h after normal culture conditions were restored. The levels of nitric oxide synthase in 50 µL of cell lysate from each well was measured according to the instructions of the TNOS Assay Kit (KGT020, KeyGEN), and then, the absorbance was measured at OD_540 nm_ using a multifunctional microplate reader. Detection of NO levels in cells by NO fluorescent probes. Briefly, 30 µg mL^−1^ nanoparticles were added to 96‐well plates (1 mm nitric oxide scavenger carboxy‐PTIO (cPTIO) was added as a negative control), and the cells were subjected to OGD/R treatment. After 6 h of reoxygenation the medium was removed, and the cells were gently washed three times with PBS. Then, 5 µm nitric oxide fluorescent probe DAF‐FMDA (excitation wavelength and emission wavelength of 490 and 520 nm, respectively) was incubated with the cells for 20 min, and the fluorescence intensity was measured with an inverted fluorescence microscope and a multifunctional microplate reader.

### Evaluation of Mitochondrial Complex 1 Activity and mPTP Opening

mPTP opening in H9C2 cells was assessed by the Calcein‐AM/CoCl_2_ quenching technique. Briefly, nanoparticles (30 µg/mL) were added to 96‐well plates containing H9C2 cells (0.1 µm cyclosporin A (CsA) was added as a negative control), and the cells were then subjected to OGD/R treatment. cPTIO (1 mm) was used to scavenge NO produced by AASP‐induced cells to further assess the effect of NO on mPTP opening. After treatment, the cells were incubated with 1 mm calcein‐AM and 2 mm CoCl_2_ for 30 min. The cells were imaged using a fluorescence microscope, and the relative fluorescence intensities were calculated. Mitochondrial respiratory chain complex I activity was determined according to the kit manual (BC0515, Solarbio). Treated cells in each group were sonicated, and mitochondria were isolated. After mixing with the corresponding substrate solution, the activity of complex I was measured spectrophotometrically.

### Fluorescence Assessment of ATP Synthase and Mitochondrial and Cellular ATP Levels

After treatment, the cells were fixed with 4% paraformaldehyde for 10 min, permeabilized with 0.1% Triton X‐100 for 5 min, and incubated with 1% bovine serum albumin at room temperature for 30 min. The cells were labelled with 5 µg mL^−1^ ATP synthase beta monoclonal antibody for 1 h, 100 nm MitoLite Red for 30 min, and 0.5 µg mL^−1^ DAPI for 5 min. The stained cells were observed, and images were captured by laser confocal microscopy. The cellular ATP levels were measured according to the Luminescent ATP Detection Assay Kit (ab113849, Abcam).

### TEM Observation of Ultrathin Sections

H9C2 cells were subjected to OGD/R according to the method described above. After treatment, the cells were harvested by centrifugation (2000 g, 5 min) and fixed overnight with 2.5% glutaraldehyde solution, followed by osmium tetroxide (1%) for 1 h. The samples were dehydrated with different concentrations (30%, 50%, 70%, 80%, 90%, or 100%) of ethanol, followed by resin embedding, sectioning, and staining with uranyl acetate solution (1%) and lead citrate solution (1%) for 5–10 min. The samples were fixed on copper grids, and the microstructures of the cells before and after treatment were observed by transmission electron microscopy.

### Myocardial Ischaemia‒Reperfusion Injury (MI/RI) Model in Rats

The animal procedures were conducted in strict accordance with the recommendations in the Guide for the Care and Use of Laboratory Animals published by Weifang Medical University. The protocols were approved by the Committee on the Ethics of Animal Experiments of Weifang Medical University (Permit Number:2020SDL183). Fifty male SD rats (300 g±20 g, 8–10 weeks old) were randomly divided into five groups: 1) sham‐operated group (Sham), 2) myocardial ischemia‒reperfusion injury group (MI/RI), 3) AS treatment group, 4) AAS treatment group, and 5) AASP treatment group. The rat myocardial ischemia/reperfusion model was established according to a previously reported method with slight modifications.^[^ ^27^ ^]^ Briefly, the SD rats were continuously anaesthetized during surgery using isoflurane. Under aseptic conditions, the heart was exposed by left open thoracotomy, acute myocardial ischemia was achieved by temporal ligation of the left anterior descending (LAD) coronary artery for 30 min, and reperfusion injury was achieved by loosening the ligature 30 min later. The rats were treated with saline (MI/RI group), AS, AAS and AASP (dose: 3 mg kg^−1^) by tail vein injection. One dose was injected immediately after surgery, and one dose was injected every two days after 24 h of reperfusion for a total of two weeks of treatment. Therefore, a total of 8 injections were administered over a 2‐week period. Blood samples were collected before and after surgery, and the plasma lactate dehydrogenase and creatine kinase isoenzyme levels were measured.

### In Vitro and In Vivo Photoacoustic Imaging

Gold nanocages were not only used as a nanodrug delivery system but also exhibit near‐infrared PA imaging properties due to the surface plasmon resonance effect.^[^ ^28^ ^]^ For in vitro PA imaging, Different concentrations of AASP (0.1 and 0.5 mg mL^−1^) were dispersed in deionized water and the PA signal was detected using a Vevo LAZR‐X (808 nm, Visual Sonics, Canada) photoacoustic imaging system. For in vivo PA imaging, the MI/RI model rats were injected with 3 mg kg^−1^ of AAS and AASP via the tail vein. PA imaging was performed using the Vevo LAZR‐X photoacoustic imaging system on rat heart cross‐sections at 0, 2, 8, and 24 h post‐injection to observe and calculate PA signal intensity in the anterior wall region of the left ventricle.

### Echocardiographic Assessment of Myocardial Function

All rats underwent echocardiography using an echocardiography system (VINNO 6VET) that was equipped with an X6‐16L transducer (6.5–18 MHz) after MI/RI and within 2 weeks after treatment. M‐mode imaging was performed in the long‐axis view of the left ventricle. LV‐EF, LV‐FS, LVIDD, LVEDV and LVESV were determined separately. All assays were performed randomly, and the researchers were blinded to the treatment group.

### Transcriptome Analysis

Total RNA was extracted from damaged left ventricular tissues from the sham, model and AASP treatment groups with TRIzol following the manufacturer's instructions, and then, a cDNA library was generated. After quality inspection, the Illumina NovaSeq6000 platform was used for PE150 sequencing. Differentially expressed gene (DEG) analysis was performed by using the edgeR package. A false discovery rate (FDR)‐adjusted *P* value≤0.05 was used as the threshold. The GO terms and KEGG pathways that met this condition were defined as the GO terms and the KEGG pathways that were significantly enriched in differentially expressed genes, respectively.

### Western Blotting

The injured left ventricular tissues of the rats in the sham‐operated group, model group and treatment group were lysed and homogenized with RIPA buffer supplemented with 1 mm PMSF. The protein concentration was determined using the BCA protein detection kit, and samples containing equal amounts of protein (40 µg lane) were loaded into SDS polyacrylamide gels. After electrophoresis, the samples were transferred to PVDF membranes. Blocking, incubation with primary and secondary antibodies, and visualization of target protein bands on the membrane were performed using ECL Western Blot Detection Reagent. Protein expression was quantified by Quantity‐One Software, and the relative expression is indicated under each band.

### Histology and Immunohistochemistry

After 2 weeks of injection treatment, all the rats were sacrificed, and heart tissues were collected. The hearts were perfused with PBS, embedded in optimum cutting temperature (OCT) compound, and immediately snap frozen in liquid nitrogen. Ten consecutive frozen sections from the left ventricular ligation site to the apex of the left ventricle were prepared at thicknesses of 10 µm with a freezing microtome (HM525 NX, Thermo Fisher Scientific). All sections were stored at −20 °C until they were stained. To assess the size of the injured area, tissue sections were fixed with 4% paraformaldehyde for 2 h and stained with Masson's trichrome. Apoptosis in the myocardial tissue region was analyzed using the DeadEnd™ Fluorescent TUNEL System (G3250, Promega). Immunohistochemistry and immunofluorescence were conducted to examine the expression of active caspase‐3, CD34, Ki67 and Ser1177‐eNOS. All images were captured with a Nikon Ti‐E inverted fluorescence microscope.

### Metabolism and Safety Assessment

To quantify nanomaterial biodistribution, SD rats were injected with 3 mg kg^−1^ AASP nanoparticles via the tail vein. The rats were sacrificed at 4, 8, 12, and 24 h, 7, 14 and 21 d after administration. The major organs, such as the heart, liver, spleen, lung, and kidney, were excised and homogenized by digestion, and the contents of Se ions and Au ions in the samples were analyzed by inductively coupled plasma‒mass spectrometry (ICP‒MS; 7500, Agilent).

The erythrocytes in 5 mL of fresh human blood were collected by centrifugation at 1000 × g for 10 min, and 5% erythrocyte stock solution (80 µL) and different concentrations of AASP solution (20 µL) were added and incubated for 1 h at 37 °C. Haemolysis was measured at OD_405 nm_ using a multifunctional microplate reader. Solutions of 0.1 M PBS buffer and acetic acid (HAc) were used as the negative and positive controls, respectively.

The in vivo toxicity caused by AS, AAS and AASP in SD rats was evaluated. Briefly, AS (3 mg kg^−1^), AAS (3 mg kg^−1^) and AASP (3/9 mg kg^−1^) were injected once daily at a fixed time via the tail vein, and an equal amount of saline was injected as a control. After 21 d of continuous administration, blood was collected from the retro‐orbital sinus and analyzed by a fully automated hematology analyzer (LH750, Beckman Coulter) to measure the levels of biochemical parameters. The hearts, livers, spleens, lungs, kidneys, and brains of the rats were collected and stained with H&E for histopathological observation.

### Statistical Analysis

All the experiments were performed at least three times, and the results are expressed as the mean ± S.D. The statistical analysis was performed using OriginPro 8.0, followed by a Student's *t*‐test and one‐way analysis of variance (ANOVA). *p < 0.05 and **p < 0.01 indicated significant differences and very significant differences, respectively.

## Conflict of Interest

The authors declare no conflict of interest.

## Supporting information

Supporting Information

## Data Availability

The data that support the findings of this study are available from the corresponding author upon reasonable request.
