# Supplementary material for: L‐Arginine‐Loaded Gold Nanocages Ameliorate Myocardial Ischemia/Reperfusion Injury by Promoting Nitric Oxide Production and Maintaining Mitochondrial Function
Source: Adv Sci (Weinh). 2023 Jul 14;10(26):2302123. doi: 10.1002/advs.202302123 (PMC10502842; doi:10.1002/advs.202302123)
Supplement: Supplementary file 1 — Supporting Information [file ADVS-10-2302123-s001.pdf]

ADVANCED SCIENCE
Open Access

Supporting Information

for *Adv. Sci.*, DOI 10.1002/advs.202302123

L-Arginine-Loaded Gold Nanocages Ameliorate Myocardial Ischemia/Reperfusion Injury by Promoting Nitric Oxide Production and Maintaining Mitochondrial Function

Zekun Wang, Nana Yang, Yajun Hou, Yuqing Li, Chenyang Yin, Endong Yang, Huanhuan Cao, Gaofei Hu, Jing Xue, Jialei Yang, Ziyu Liao, Weiyun Wang, Dongdong Sun, Cundong Fan* and Lemin Zheng**

ADVANCED SCIENCE
Open Access

Supporting Information

for *Adv. Sci.*, DOI 10.1002/advs.202302123

L-Arginine-Loaded Gold Nanocages Ameliorate Myocardial Ischemia/Reperfusion Injury by Promoting Nitric Oxide Production and Maintaining Mitochondrial Function

Zekun Wang, Nana Yang, Yajun Hou, Yuqing Li, Chenyang Yin, Endong Yang, Huanhuan Cao, Gaofei Hu, Jing Xue, Jialei Yang, Ziyu Liao, Weiyun Wang, Dongdong Sun, Cundong Fan* and Lemin Zheng**

Supporting Information

for

L-Arginine-Loaded Gold Nanocages Ameliorate Myocardial Ischaemia/Reperfusion Injury by Promoting Nitric Oxide Production and Maintaining Mitochondrial Function

Zekun Wang[1], Nana Yang[2], Yajun Hou[3], Yuqing Li[1], Chenyang Yin[1], Endong Yang[1], Huanhuan Cao[4], Gaofei Hu[4], Jing Xue[5], Jialei Yang[5], Ziyu Liao[1], Weiyun Wang[1], Dongdong Sun[1*], Cundong Fan[3*], Lemin Zheng[4,5*]

[1] School of Life Sciences, Anhui Agricultural University, Hefei, Anhui, 230036, China

[2] School of Bioscience and Technology, Weifang Key Laboratory of Animal Model Research on Cardiovascular and Cerebrovascular Diseases, Weifang Medical University, Weifang 261053, China

[3] Department of Neurology, Second Affiliated Hospital, Shandong First Medical University & Shandong Academy of Medical Sciences, Taian 271000, Shandong, China

[4] The Institute of Cardiovascular Sciences and Institute of Systems Biomedicine, School of Basic Medical Sciences, Key Laboratory of Molecular Cardiovascular Sciences of Ministry of Education, Health Sciences Center, Peking University, Xueyuan Road NO.38, Haidian District, Beijing 100871, China

[5] Department of Neurology, China National Clinical Research Center for Neurological Diseases, Beijing Tiantan Hospital, Capital Medical University, Beijing, 100070, China

[a] Zekun Wang and Nana Yang contributed equally to this work.

***Corresponding author:**

Lemin Zheng, Tel: +86 010 82805452, Fax: +86 010 82805452, Email: zhengl@bjmu.edu.cn

Cundong Fan, Tel:: +86 0538-6230027, Fax: +86 0538 6230027, Email: cdfan@sdfmu.edu.cn

Dongdong Sun, Tel: +86-0551-65786703; Fax: +86-0551-65786703. Email: sunddwj@ahau.edu.cn

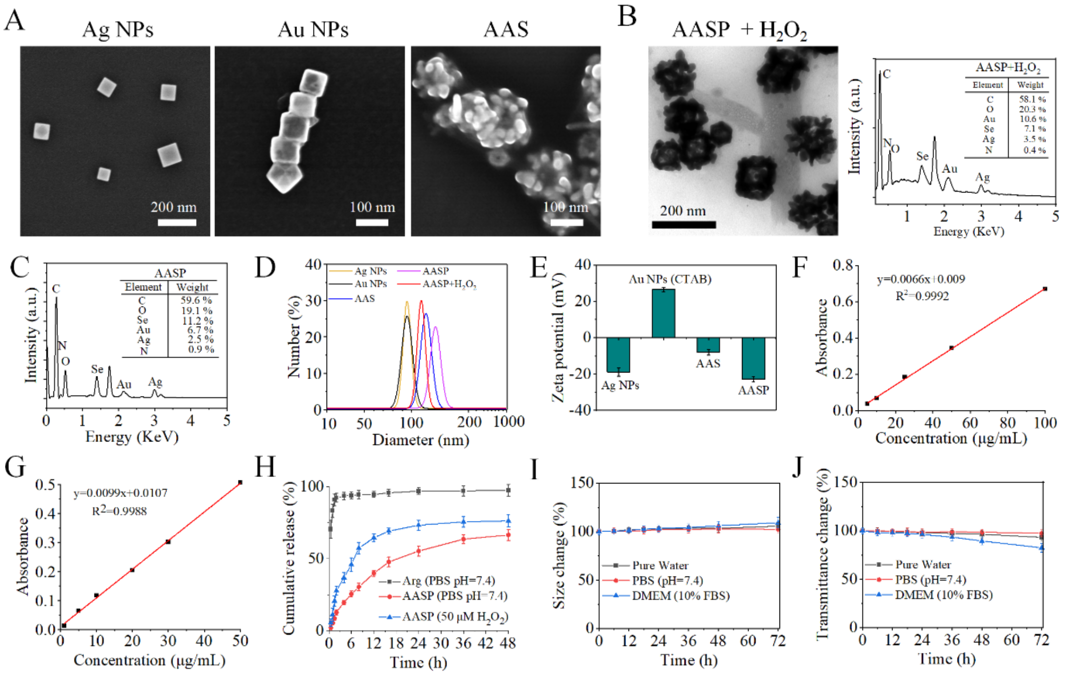

Figure S1. (A) SEM images of Ag NPs, Au NPs and AAS. (B) TEM imaging and elemental analysis after incubation with AASP and H_2O_2. (C) Elemental analysis of AASP and weight percentages of each element. Particle size distribution (D) and Zeta potential (E) of Ag NPs, Au NPs, AAS and AASP. Standard curves and regression equations for L-Arg (F) and PCM (G). (H) Release rates of L-Arg from AASP in different solutions within 48 h. The black line indicates that the retention effect of the dialysis bag itself has no significant effect on the release of L-Arg alone. Thermodynamic stability (I) and Kinetic stability (J) of AASP in pure water, PBS or DMEM medium containing 10% FBS.

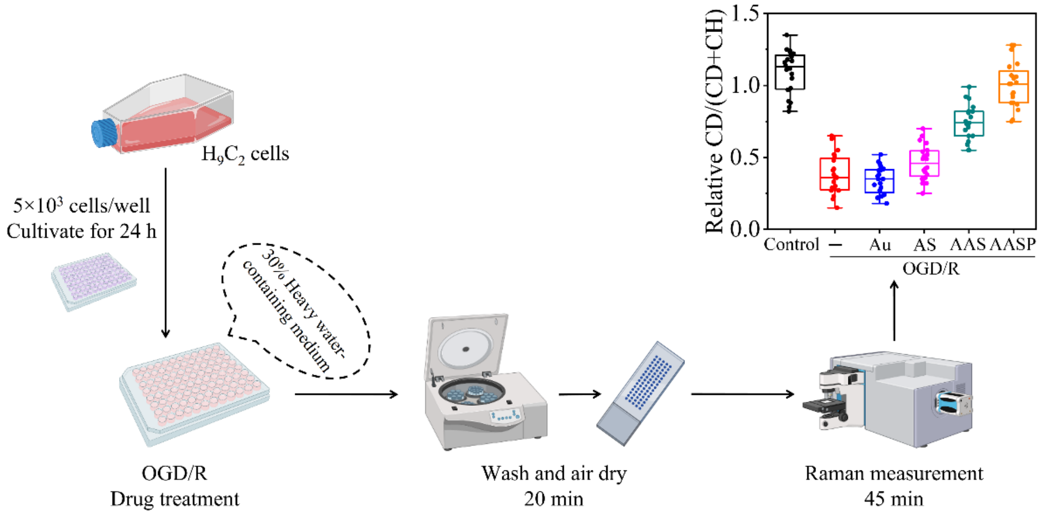

Figure S2: Single-cell Raman spectra of H9C2 cells after different treatments. Calculate the CD/(CD+CH) ratio to present the general metabolic activity of the cells.

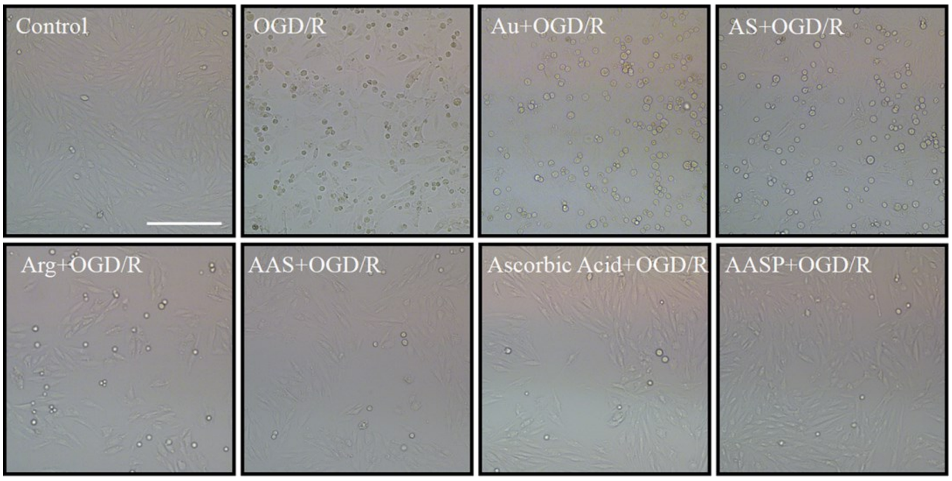

Figure S3. White light images of H9C2 cells treated with OGD/R, OGD/R+Au, OGD/R+AS, OGD/R+Arg, OGD/R+AAS, OGD/R+ascorbic acid and OGD/R+AASP, respectively. Scale bar = 50 μm.

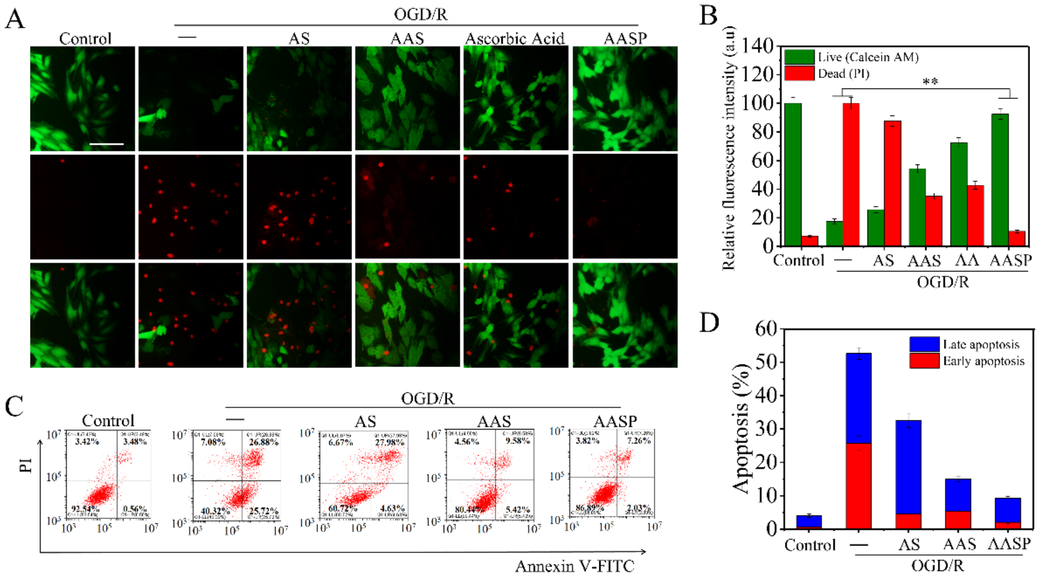

Figure S4. (A) Fluorescence microscopy images and relative fluorescence intensities (B) of H9C2 cells co-stained with Calcein-AM (Live)/PI (Dead) under different treatments. Scale bar = 50 μm. (C) Flow cytometry detection of apoptosis (Annexin V-FITC/PI) after different treatments. (D) Statistical analysis of early apoptosis and late apoptosis of cells after different treatments. The data are expressed as the mean ± SD, **P* < 0.05, ***P* < 0.01.

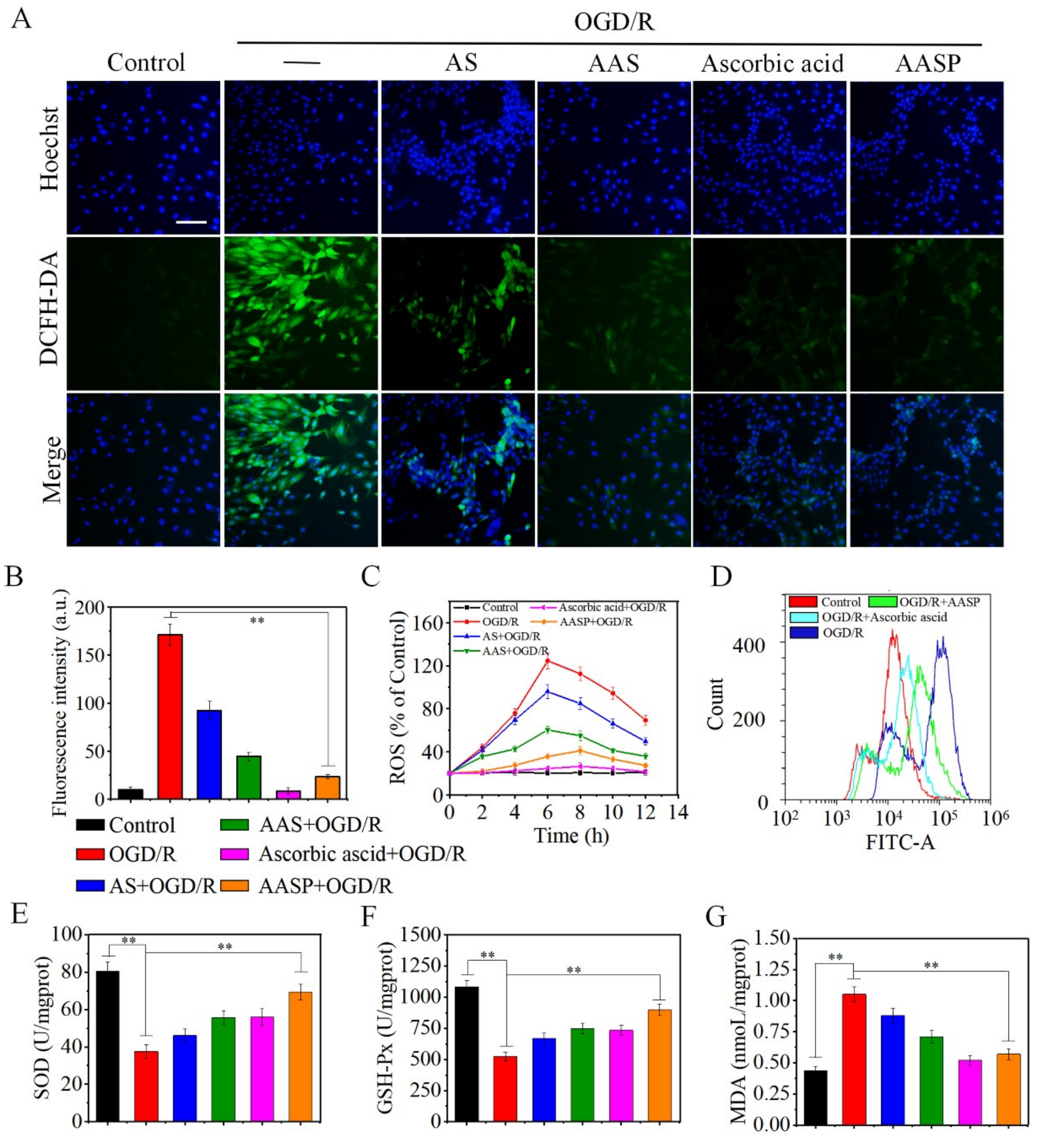

Figure S5. (A) ROS intensity of H9C2 cells in different treatments was detected with DCFH-DA. Scale bar = 50 μm. (B) Relative fluorescence intensity in cells of different treatments. (C) Changes in ROS levels over time in cells with different treatments. (D) Analysis of ROS levels in differently treated cells by flow cytometry. The contents of SOD (E), GSH-Px (F) and MDA (G) in different treated cells were detected at the cellular level. The data are expressed as the mean ± SD, *$P < 0.05$, **$P < 0.01$.

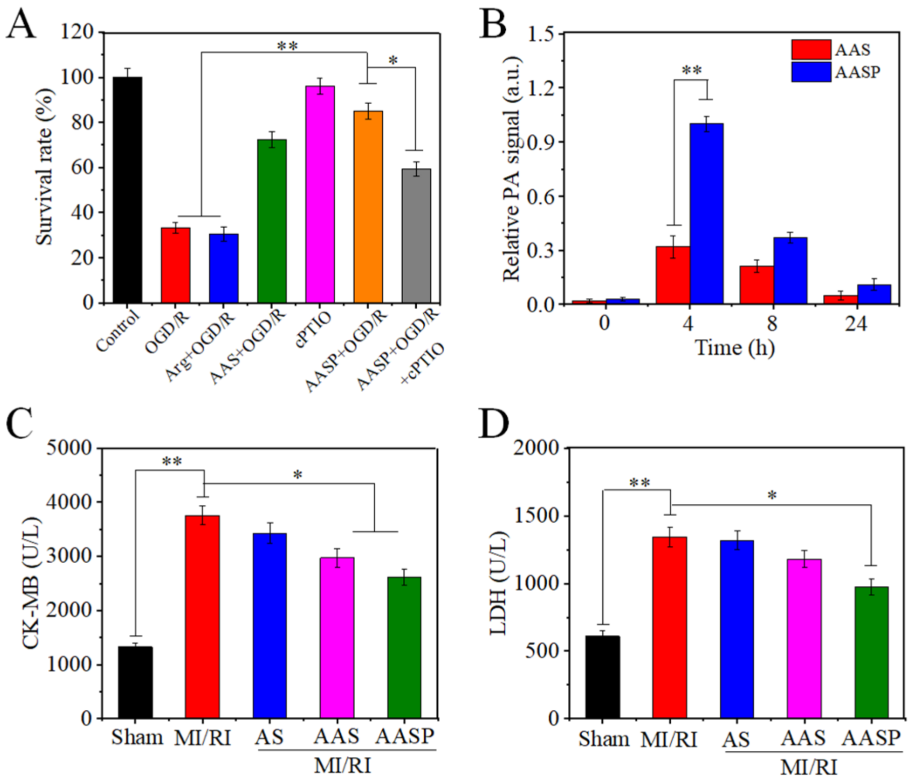

Figure S6. (A) Effect of NO on cell viability after OGD/R treatment. (B) The PA signal intensity of AAS or AASP was detected in the left ventricle at 0h, 4h, 8h and 24h after tail vein injection of AAS or AASP, respectively. The levels of CK-MB (C) and LDH (D) in the serum of rats in each group were detected 2 h after MI/RI modeling. The data are expressed as the mean ± SD, *$P < 0.05$, **$P < 0.01$.

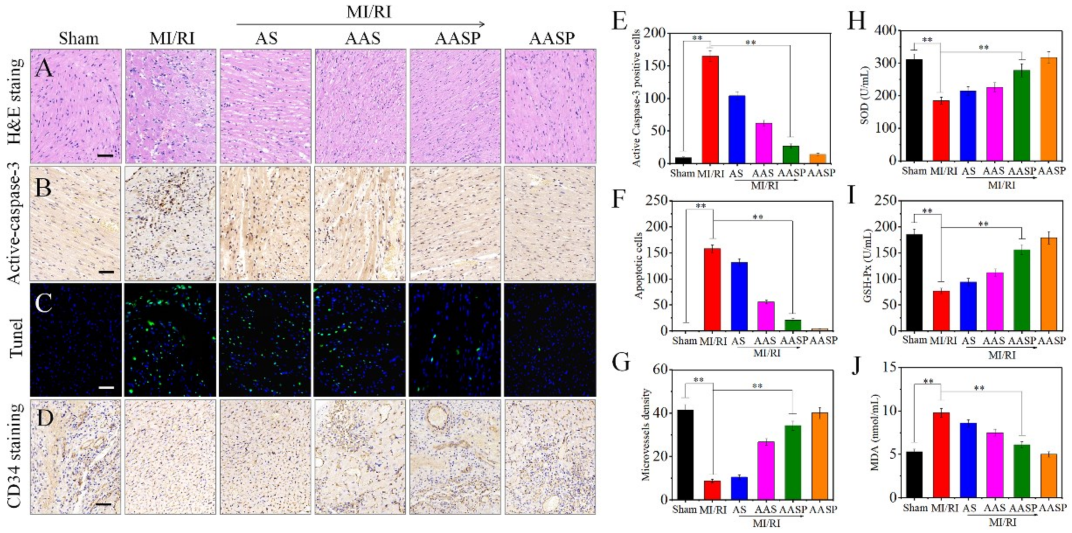

Figure S7. (A) H&E staining was used to analyze the histopathology of MI/RI regions in the sham-operated group, model group, AS-treated group, AAS-treated group and AASP-treated group. Scale bar = 50 µm. Active-caspase-3 (B) and Tunel immunofluorescence staining (C) detected the level of apoptosis in the MI/RI region. Scale bar = 50 µm. Quantitative analysis of Active-caspase-3 positive cells (E) and Tunel positive cells (F). (D) CD34 labelling of vascular endothelial cells in the MI/RI areas of rats and (G) quantitative assessment of microvessel density. Scale bar = 50 µm. Detection of SOD (H), GSH-Px (I) and lipid peroxide MDA (J) in serum of rats in each treatment group. The data are expressed as the mean ± SD, n = 10 animals per group, *$P < 0.05$, **$P < 0.01$.

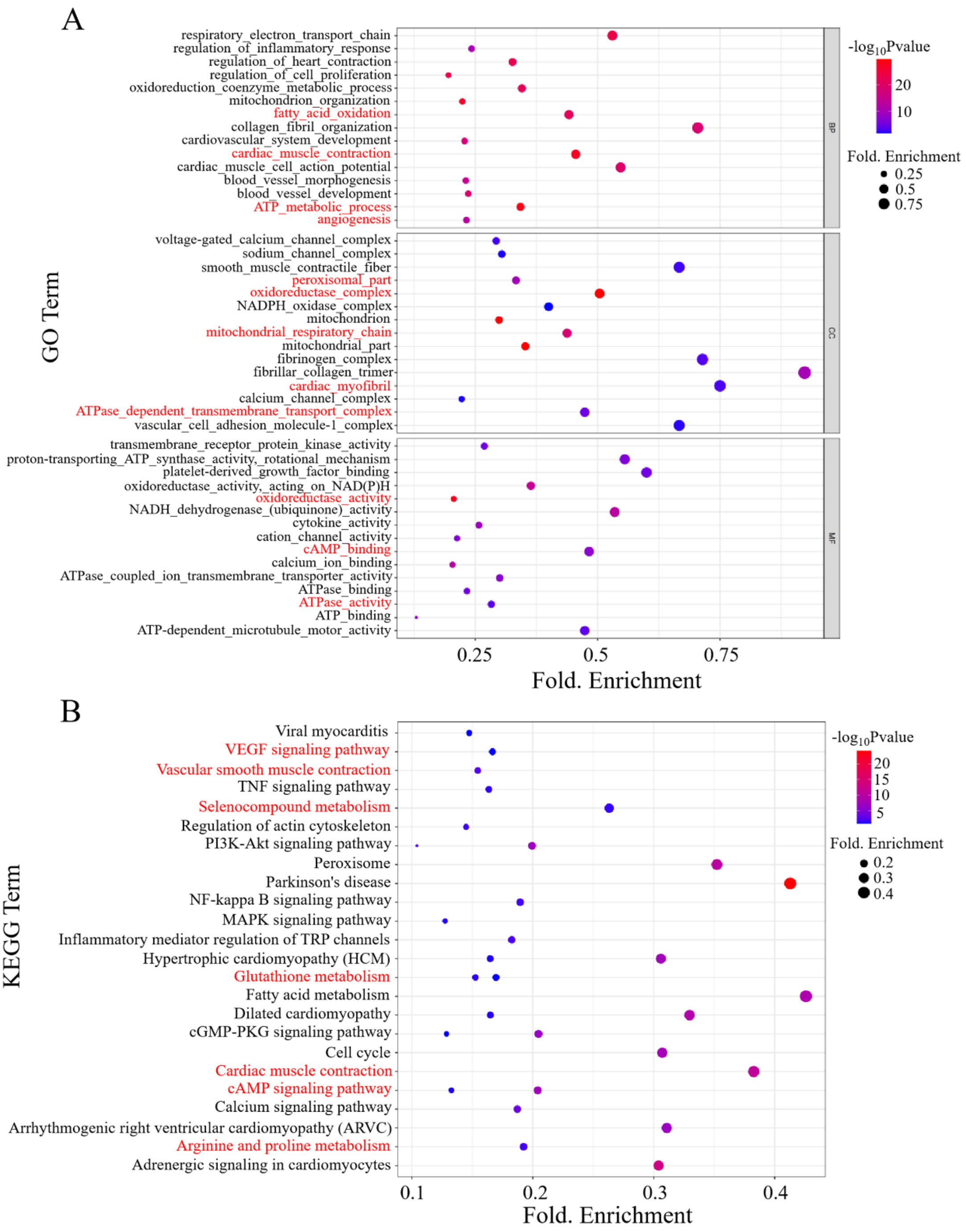

Figure S8. (A) GO enrichment analysis of DEGs in the damaged area of the myocardium in the model group and the AASP-treated group. (B) KEGG pathway enrichment analysis was performed on DEGs in the damaged myocardial region in the model group and the AASP-treated group. Important paths are indicated in red font.

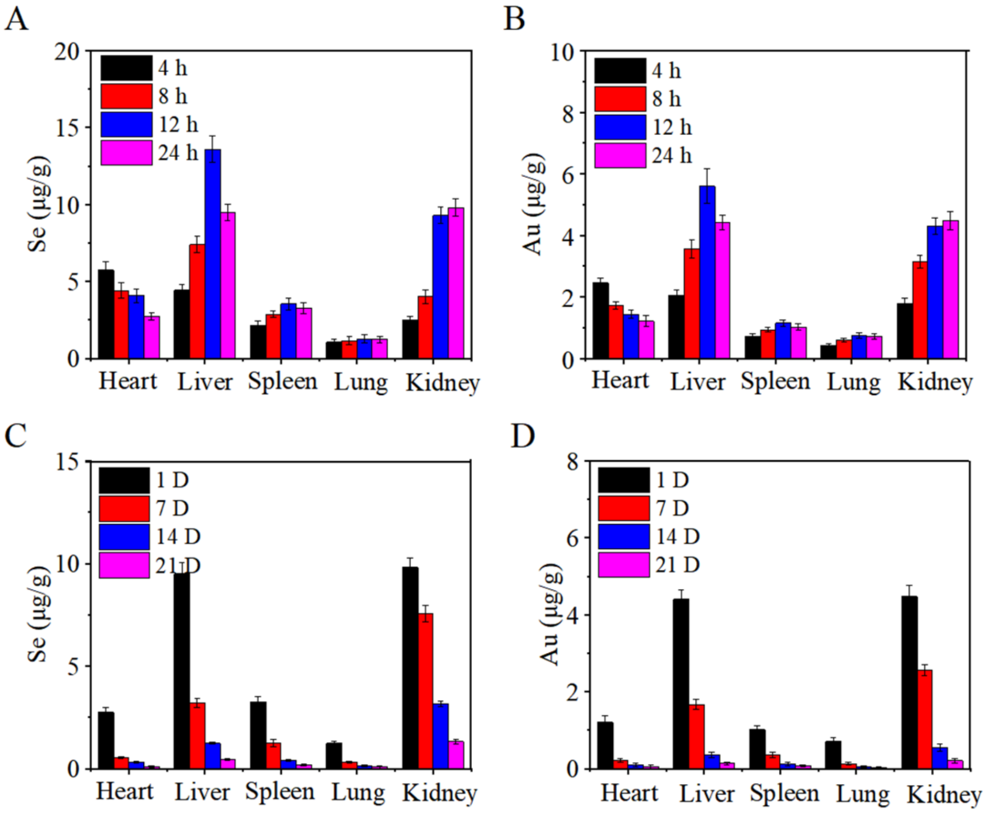

Figure S9. AASP was injected through the tail vein of rats, and the main organs were collected at 4h, 8h, 12h and 24h after injection, and the residual Se (A) and Au (B) in each organ were determined. To detect the *in vivo* metabolism of AASP for a long time, the main organs were collected on the 1st, 7th, 14th and 21st days, respectively, and the residual Se (C) and Au (D) in each organ were determined.

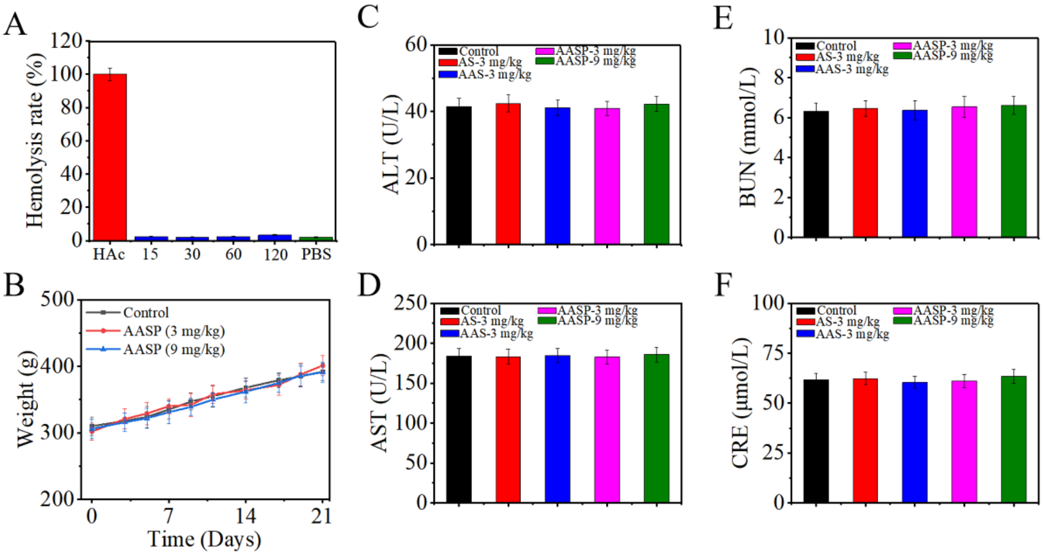

Figure S10. (A) Hemolytic activity of AASP (15, 30, 60 and 120 μg/mL). (B) Effect of 3 mg/kg or 9 mg/kg of AASP on body weight in rats over 21 days. Effects of 3 mg/kg or 9 mg/kg of AASP on the blood levels of ALT (C), AST (D), BUN (E) and CRE (F) in rats after 21 days of administration via tail vein.

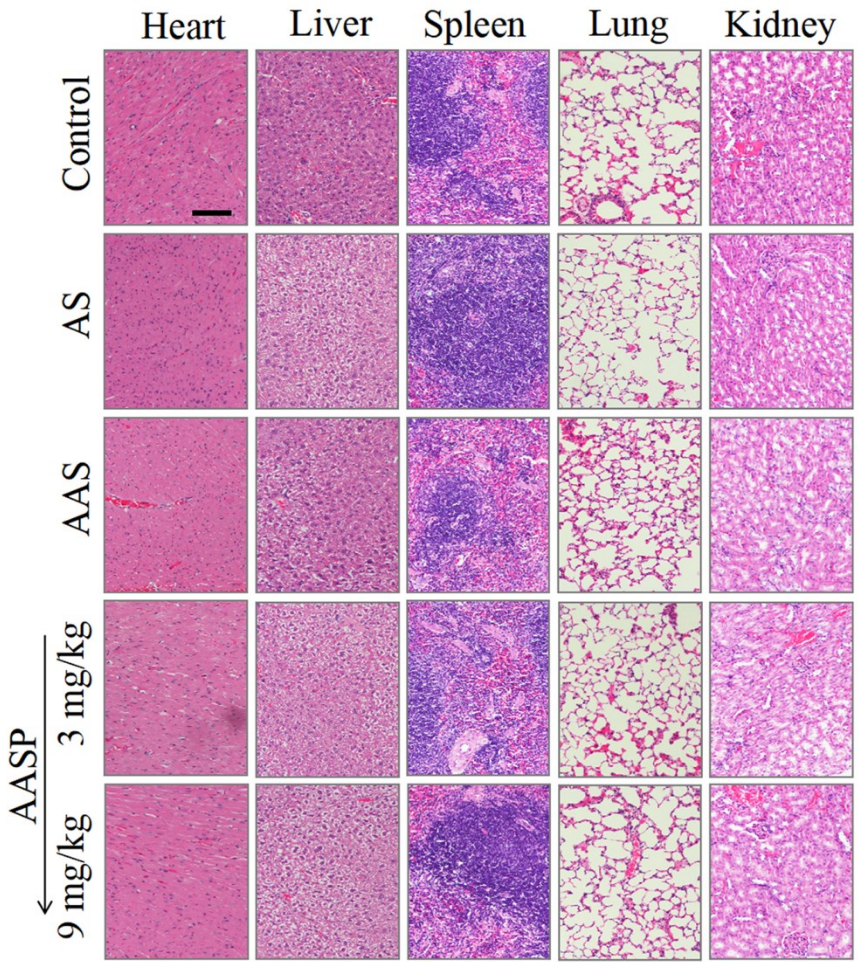

Figure S11. The heart, liver, spleen, lung and kidney were detected by H&E staining on the 21st day after the tail vein injection of AS (3 mg/kg), AAS (3 mg/kg) or AASP (3/9 mg/kg). Scale bar = 50 μm.
